# Impact of Cryopreservation on Spermatozoa Freeze-Thawed Traits and Relevance OMICS to Assess Sperm Cryo-Tolerance in Farm Animals

**DOI:** 10.3389/fvets.2021.609180

**Published:** 2021-02-25

**Authors:** Ibrar Muhammad Khan, Zubing Cao, Hongyu Liu, Adnan Khan, Sajid Ur Rahman, Muhammad Zahoor Khan, Anucha Sathanawongs, Yunhai Zhang

**Affiliations:** ^1^Anhui Provincial Laboratory of Local Livestock and Poultry Genetical Resource Conservation and Breeding, College of Animal Science and Technology, Anhui Agricultural University, Hefei, China; ^2^Shenzhen Branch, Guangdong Laboratory for Lingnan Modern Agriculture, Genome Analysis Laboratory of the Ministry of Agriculture, Agriculture Genomics Institute at Shenzhen, Chinese Academy of Agricultural Sciences, Shenzhen, China; ^3^Key Laboratory of Animal Parasitology of Ministry of Agriculture, Laboratory of Quality and Safety Risk Assessment for Animal Products on Biohazards (Shanghai) of Ministry of Agricultural Sciences, Shanghai Veterinary Research Institute, Chinese Academy of Agricultural Sciences, Shanghai, China; ^4^State Key Laboratory of Animal Nutrition, Beijing Engineering Technology Research Center of Raw Milk Quality and Safety Control, College of Animal Science and Technology, China Agriculture University, Beijing, China; ^5^Department of Veterinary Biosciences and Veterinary Public Health, Faculty of Veterinary Medicine, Chiang Mai University, Chiang Mai, Thailand

**Keywords:** spermatozoa cryo-biology, functional traits, cryo-injuries, cryo-tolerance fingerprints, molecular tools

## Abstract

Sperm cryopreservation is a powerful tool for the livestock breeding program. Several technical attempts have been made to enhance the efficiency of spermatozoa cryopreservation in different farm animal species. However, it is well-recognized that mammalian spermatozoa are susceptible to cryo-injury caused by cryopreservation processes. Moreover, the factors leading to cryo-injuries are complicated, and the cryo-damage mechanism has not been methodically explained until now, which directly influences the quality of frozen–thawed spermatozoa. Currently, the various OMICS technologies in sperm cryo-biology have been conducted, particularly proteomics and transcriptomics studies. It has contributed while exploring the molecular alterations caused by cryopreservation, identification of various freezability markers and specific proteins that could be added to semen diluents before cryopreservation to improve sperm cryo-survival. Therefore, understanding the cryo-injury mechanism of spermatozoa is essential for the optimization of current cryopreservation processes. Recently, the application of newly-emerged proteomics and transcriptomics technologies to study the effects of cryopreservation on sperm is becoming a hotspot. This review detailed an updated overview of OMICS elements involved in sperm cryo-tolerance and freeze-thawed quality. While also detailed a mechanism of sperm cryo-injury and utilizing OMICS technology that assesses the sperm freezability potential biomarkers as well as the accurate classification between the excellent and poor freezer breeding candidate.

## Introduction

Sperm cryopreservation has become a popular technique for the long-lasting semen preservation of genetically superior animals, related transgenic lines, and mammalian endangered species (1, 2). Besides, cryopreservation assists the wide spread of genetic diversity, and contributed greatly into the extension of reproductive technologies worldwide, such as artificial insemination and *in-vitro* fertilization (3).

However, cryopreservation can have a detrimental effect on the normal physiology of sperm, causing damage and modifications that eventually lead to the death of the sperm, thereby reducing freeze-thawed quality parameters (2). Furthermore, the conflicts in sperm size, shape, and lipid-protein content among the species demonstrate that cryopreservation methods are not fairly efficient in all species (4). It has been recorded by Grötter et al. (5) that farm animals like bull, ram, and boar produce more cryo-sensitive spermatozoa than human, rabbit, cat, and dog. In addition to the interspecies variability, many other variables such as freezing-thawing rates, type of semen extenders or cryo-protectants, the origin of spermatozoa (epididymal or ejaculate sperm), seasonal fluctuations, and even inter-or intra-individual variations also influence the success of the cryopreservation method (6, 7).

In 1937, glycerol was used as freezing medium for semen of bull, ram, stallion, boar, and rabbit at cooling (−21°C) phase. The good cryo-protective effects were obtained when the glycerol concentrations ranged from 0.5 to 2 M (8). Then, about 10 years later, the Polge et al. (9) further confirmed the positive effects of glycerol on frozen poultry semen. However, the glycerol causes toxicity in sperm by denaturation of protein, alteration *via* actin interactions, and induction of plasma membrane fragility during cryopreservation (9–11). Another significant breakthrough was achieved during the 1950s, when dry ice was replaced by liquid nitrogen as a freezing medium; since sperm can be preserved viable at −196°C unlimitedly. On the contrary, dry ice cannot completely stop the metabolic activity of mammalian cells (12). However, it should be noted that some drawbacks still exist about the concept whether storage in liquid nitrogen is completely harmless to the viability of frozen sperm (13, 14).

Impact of cryopreservation on sperm biology produced novel consequences; and has led to the development of modern cryopreservation techniques where particular proteins, antioxidants, and cryo-protective agents are integrated into the freezing medium to enhance the cryo-survival of spermatozoa (15). There has been no genetic selection of the breeding stocks for semen cryopreservation in animal breeding programs, even though improvement has been found in outlining the major genes involved in spermatozoa cryo-biological function (16). Although it has been proved that some sperm protein markers are correlated with high cryo-tolerance, their function is reliant on the presence of mRNA (7). It has been recommended that spermatozoa RNAs evaluation provides valuable information on their biological function (16, 17).

However, to date, there is a limited collection of literature about the associations of OMICS with spermatozoa freeze-thawed quality of farm animals. The spermatozoa freeze-thawing resilience varies based on their physical characteristics, such as size, shape, and lipid content. Therefore, it is difficult to establish a standardized freezing technique for the breeding management in various species of animal. The review explored how cryopreservation alters the structural and molecular integrity of freeze-thawed spermatozoa. Additionally, the review also details the current understanding of the OMICS element present in the farm animal spermatozoa and their potential use in predicting sperm cryo-tolerance.

## Cryopreservation Deteriorates Spermatoza Freeze-Thawed Quality

**Figure 1:**
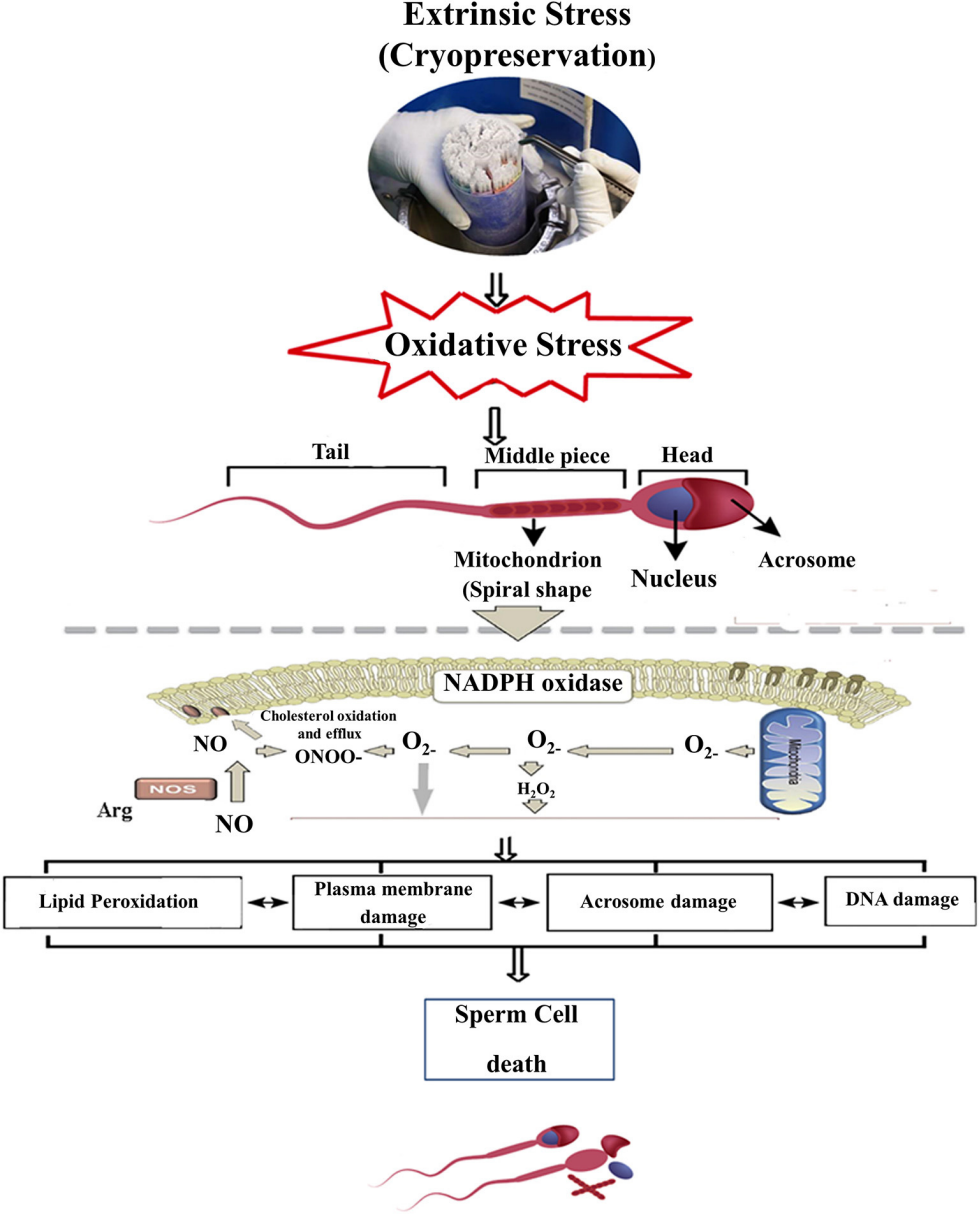
Scheme represents cryopreservation damages in the sperm cell, whereas an excess induction of oxidative stress in resulting ROS production can deteriorate the sperm plasma membrane and acrosomal membrane and eventually alter the molecular structure (DNA). ROS, Reactive oxygen species; H_2_O_2_, Hydrogen peroxide; ^•^${\text{O}}_{2}^{-}$, Superoxide radical; NADPH, Nicotinamide adenine dinucleotide phosphate; ONOO^−^, Peroxy nitrate, NO; Nitric oxide.

Cryopreservation damages the sperm in a variety of ways such as ultra-structural damage and sub-lethal damages that encourage oxidative and osmotic stresses, which amend lipid and protein configuration, decrease motility and viability, cause injury to mitochondria and spermatozoa tail, and intensify sperm DNA fragmentation, leading to a decline in freeze-thawed sperm quality as shown in Figure 1 (2, 18). A spermatozoon consists of several membranes, such as plasma membrane, mitochondrial membrane, and the acrosomal membrane. These membranes act as physiological barriers that must remain intact to ensure sperm viability, particularly after cryopreservation (13, 19). Cryopreservation induces structural damages of mitochondria, altering the biochemical processes involved in ATP production and ultimately reducing spermatozoa freeze-thawed viability and motility (20).

### Structural and Molecular Integrity of Freeze-Thawed Spermatozoa

The spermatozoa plasma membrane is the midline between the inner and outer environments. The plasma membrane plays a vital role for male and female gametes, displaying receptors responsible for sperm–oocyte interactions (21). Integrity of membrane-intact spermatozoa is required for survival in the female genital tract. Alterations in membrane structures may be associated with dysregulation of the lipids, resulting in oxidative stress (22). The higher ratio of unsaturated to saturated fatty acid in the plasma membrane makes more susceptible to cryopreservation-related damage and peroxidation (23). More damage has been detected in the plasma membrane and acrosome membranes during freezing-thawing cycle because these parts are more exposed to cryo-environment and thus suffering from ultra-structural biochemical and functional changes. These changes inhibit spermatozoa movement in the female reproductive tract, reducing fertility in animal species (24).

The structural and functional integrity of the spermatozoa acrosome is considered necessary to attain high fertility; however, cryopreservation can damage the acrosomal layer, diminishing the ability of spermatozoa to penetrate the zona pellucida (25). Cryopreservation can affect the acrosomal membrane and induce a pre-acrosomal reaction, thus influencing the viability and quality of the spermatozoa. Sperm freeze-thawing induces capacitation, and sudden occurrence of acrosome reaction-like changes in mammalian spermatozoa (1, 2). The acrosomal reaction further assists the sperm to achieve fertilization, hence sperm cell quality is evaluated based on proper capacitation, acrosomal reaction, regular fertilization, and early embryonic development (26).

Spermatozoa DNA integrity is considered very important because it protects the genetic material and transfers the paternal characteristics into offspring, It has been found that damaged DNA may harm fertilization, embryogenesis, and the healthy live birth rate in mammals (27). Spermatozoa DNA disintegration is characterized by single and double-stranded DNA breaks, which occur during or after DNA wrapping; some of these breaks might escape the DNA repairing mechanism and be transferred into mature spermatozoa. Aberrant spermatozoa chromatin packaging, oxidative stress, and abortive apoptosis are the etiological factors that lead to DNA strand breaks (28, 29). For successful fertilization after sperm penetrates the oocyte, the spermatozoa chromatin material must undergo de-condensation (30). Cryopreservation can damage spermatozoa DNA integrity, influencing the sperm functional potential and the successful fetal development (31).

### The Mechanism of Spermatozoa Cryo-Injury in the Cryopreservation Methods

During the cryopreservation process, the mammalian spermatozoa have to endure various types of stresses caused by ice formation, chemical toxicity, and oxidative stress, which mainly damage cytoplasm membrane, consequently leading to a lower post-thawed quality and fertility (10, 32, 33). According to the traditional theory, the cryo-damages of mammalian cells are mainly derived from ice crystal formation and chemical toxicity. However, different from other somatic cells, spermatozoa cells contain lower water content and higher protein concentration. In general, the water content in spermatozoa is ~60% and lower than that of typical somatic cells (>80%) (34). Therefore, it is presumed that the effects of ice formation on spermatozoa may be less as compared to other somatic cells. However, despite the above hypothesis, some researchers still think that it is necessary to prevent ice formation in spermatozoa. Some specific protectants, such as antifreeze proteins (35, 36) or synthetic ice blockers (37) were used to modify ice crystal shape during sperm cryopreservation. But, it should be noted that disputes related to ice formation still exist. Additionally, the sperm plasma membrane is extremely sensitive to osmotic stress. However, ice formation can aggravate the effects of osmotic stress on sperm during freezing (38). In addition to cited factors, the oxidative stress caused by cryopreservation should not be neglected, because long-time exposure to oxygen cannot be completely avoided during cooling or freezing (39–41).

When reactive oxygen species (ROS) exceeds the defense mechanisms of sperm, consequent damage occurs in the cell membrane structure and molecular modification as well. This damage can reduce post-thawed fertility of spermatozoa, and the zygotes or embryo often fail to be carried through to full-term pregnancy (42, 43). The equine spermatozoa have the potential to produce ROS, and the average level of ROS plays a vital role in the signaling events that control sperm capacitation (31, 44), spermatozoa acrosome reaction, hyper-activation, and sperm–oocyte fusion (45). High levels of ROS production can cause polypeptide chains in the spermatozoa to become fractured that may reduce ATP production, which leads to inadequate axonemal phosphorylation, increased lipid peroxidation, and loss of motility. When the equilibrium between ROS and antioxidants is disturbed, leading to malformed spermatozoa and eventually male infertility (46, 47), and it is considered the main causative factor for spermatozoa DNA damage (39). The only reactions that can occur in frozen aqueous systems at −196°C are photophysical events such as the formation of free radicals and the production of breaks in macromolecules, and these events support the damaging of sperm DNA material (48). However, the expected increase occurs in ROS production during freeze-thawing; thus cells become under rescue and facing oxidative stress. ROS manufactured as byproducts of redox reactions, it is essential for cellular function and acts as signaling agents, the stimulation of specific transcription factor-like “NF-kB and AP-1” to sustain energy metabolism and hence to rescue the cell (49). The manufacturing of ROS during spermatozoa freezing is well-reputable, although the freezing and thawing cycle altered the electron transport chain in mitochondria and oxidase NADPH in the plasma membrane (50).

## Relevance Omics Exploration and Spermatozoa Cryo-Tolerance

**Figure 2:**
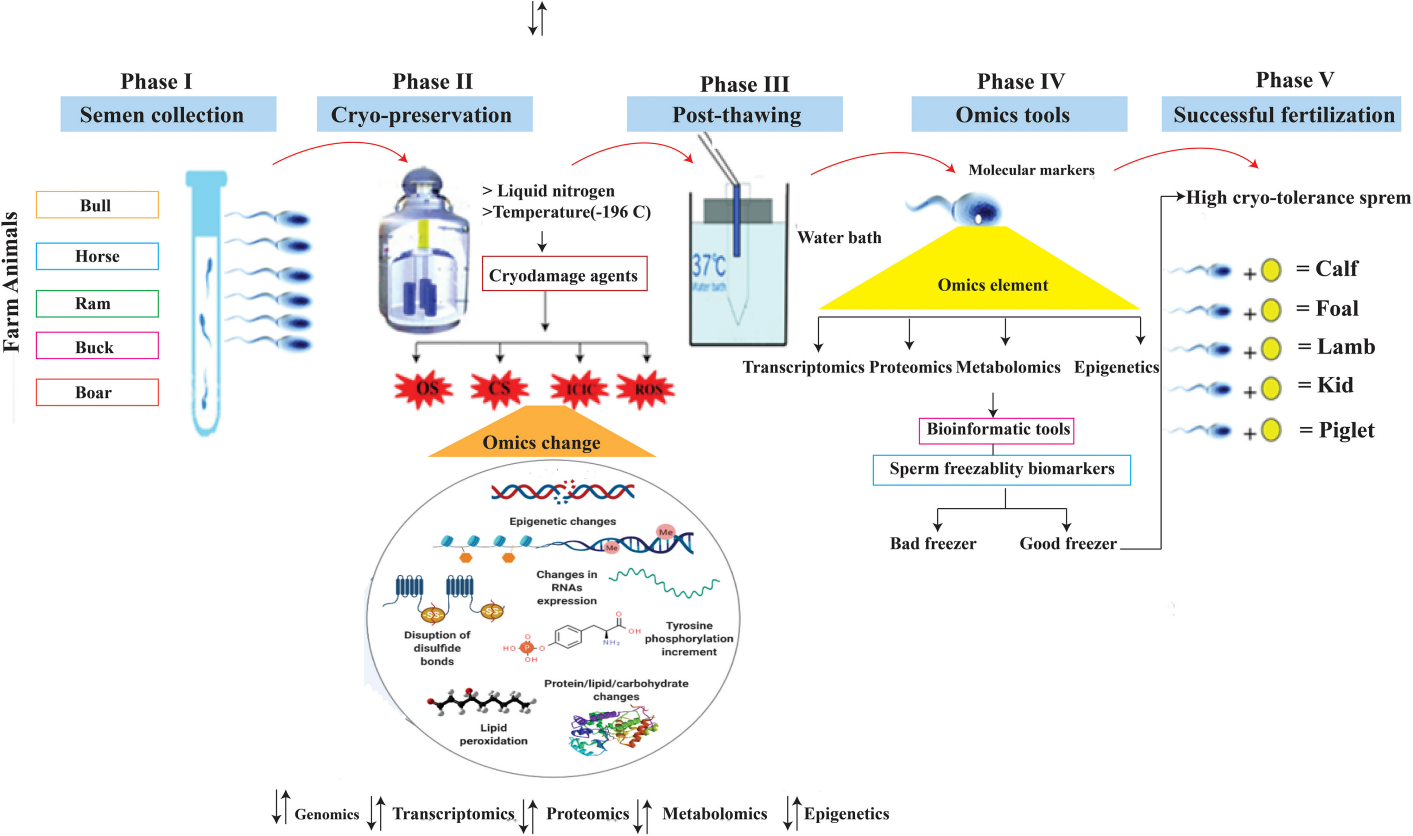
Schemes showed five important phases of sperm biotechnology: Phase I highlighted the semen collection from farm animals, phase II showed semen cryo-storage at liquid nitrogen where temperature is −196°C and produces various stresses which changed the omics elements, phase III highlighted the sperm thawing process, phase IV suggested utilization of OMICS tools for development of cryo-markers, and the last phase supposed the sperm cryo-tolerance efficacy and led to successful fertilization. OS, Osmotic stress; CS, Cold shock; ICIC, Intracellular ice crystal Formation; ROS, Reactive oxygen species.

Semen from bulls, boars, and rams were tested for motility parameters using the computer-assisted sperm analyzer (CASA) and found to be statistically significant, although there are still major variations in their ability to develop viable embryos, both *in vitro* and *in vivo* (51, 52). The transcriptome and proteome monitor the genome expression, and along with phenotypic traits and environmental knowledge provide an opportunity for a systematic OMICS approaches to understanding normal and abnormal cell biology (53). Identification and validation of OMICS biomarkers, such as genes, transcripts, proteins, and metabolites, primarily associated with seminal plasma and spermatozoa of livestock species, have a great potential to improve the reproductive efficiency of farm animals. Single nucleotide polymorphisms (SNPs) are the most frequent type of mutation in the genome, and these single base substitutions are correlated with perceived genetic features in the DNA code (54, 55). For example, nucleotide substitutions in the coding region of FSHβ, the beta-subunit follicle-stimulating hormone (FSH) gene, were associated with reduced semen quality, sperm cryosurvival, and conception rates in beef cattle (56). Metabolites such as 2-oxoglutaric acid and fructose are potential biomarkers of the quality and fertility of the frozen sperm of the bull (57). The proteome (PEBP4) also appears to be a reservoir of potential biomarkers related to bull spermatozoa—freezing and fertility (58). Increasing evidence suggests that transcriptoms such as mRNA, microRNA (miRNA), small non-coding RNAs, and piwi-interacting RNA (piRNA) may have a functional role in early embryogensis and serve as biomarkers of male reproductive performance. To that purpose, RNA sequencing (RNA-Seq) and other approaches have been used to assess the occurrence and quantity of RNA in animal freeze-thawed spermatozoa (59–61). The use of current omics technology in cellular biology is the need of the day and an excellent tool for exploring spermatozoa molecular occupation. Cryo-biology plays a crucial role in the preservation of genetics, but it can degrade the consistency of spermatozoa. The wide range of genetic variations in freezing-thawing spermatozoa has encouraged the selection of breeding animals whose semen can tolerate cryopreservation stresses (Figure 2).

### Proteomics May Provide an Opportunity for the Elucidation of Spermatozoa Cry-Tolerance

Currently, seminal plasma proteins are considered the basic units in advanced reproductive technology, and it is evident that proteins are involved in different spermatozoa biological mechanisms such as energy production, the glycolysis cycle, the citric acid cycle, and oxidative phosphorylation, which maintain the sperm in a physically active state (62). Some studies illustrate common issues regarding frozen stimulus damages of bovine spermatozoa. A former study matched protein levels in pre- and post-thawed sperm using isobaric tags for comparative and complete quantitation (iTRAQ) technology and found that variations in the identified proteins affected the quality of freeze-thawed sperm, probably decreasing the fertilizing capacity in swine (63). There are some definite spermatozoa proteomic markers of the good freezer and bad freezer animals that have been identified in domestic animals (64); a higher level of voltage-dependent anion channel 2, heat shock protein 90, and low level of triosephosphate isomerase is associated with good freezability in boar sperm (65, 66).

There is considerable variability in spermatozoa ability to withstand cryopreservation procedures between and within ejaculates. Some sperm-specific proteins have been identified as associated with the post-thawing phenomena, and their expression patterns are involved with cell resistance against freeze-thaw damage. Furthermore, the differential expression patterns of seminal plasma and sperm proteins could be developed as freezability biomarkers (63, 64, 67). Vilagran et al. (68) recognized VDAC2 as a possible positive biomarker of spermatozoa cryopreservation in swine, whereas the occurrence of VDAC2 in higher quantities in good cryo-tolerance spermatozoa suggests its contribution in the protection of spermatozoa from changes in membrane fluidity through improved regulation of ion transportation across the membrane during cold shock trials in the cryopreservation process. The higher level of fertility-linked 28-30-kDa heparin-binding proteins (HPBs) in seminal plasma enhances the conception rate by 13% while comparing to lack of these proteins, and also provides better cryo-protective support during the cryopreservation (69). It has been reported that the higher levels of fertility-linked 28–30-kDa heparin-binding proteins (*HPBs*) in semen could provide better cryo-protective support to sperm morphology and membrane integrity, achieving a 13% higher conception rate compared with that induced by semen lacking these proteins (22).

We acknowledged some enzymes in the good freezability semen that guarded sperm against oxidative stress, and it found in two forms (Rho and Pi) of glutathione S-transferase (GST) group. Hence, an enrichment of defensive intracellular proteins and membrane enzymes in spermatozoa of good freezability would be a great advantage, as these sperm cells are wide-open to ROS production during cryo-stimulus and their function could be related to enhanced protection of sperm membrane (70). Boar spermatozoa genomics analysis indicated that the protein level of outer dense fiber 2 (ODF2), heat shock protein (HSP90AA1), A-kinase-anchoring proteins 3 and 4 (AKAP3 and AKAP4), voltage-dependent anion channel 2 (VDAC2), triosephosphate isomerase 1 (TP1), and acrosin-binding protein (ACRBP), were associated with good freezability semen (63, 64). AKAP4 and AKAP3 were found in the fibrous sheath of spermatozoa flagellum and are involved in sperm motility and morphology. High expression of AKAP4 or AKAP3 in freeze-thawed spermatozoa was linked with premature capacitation (71).

Adenylate kinase isoenzyme 1 (AK1) and phosphatidylethanolamine-binding protein 1(PEBP1) were found abundantly in bull, horse, and boar spermatozoa with higher cryo-associated rates. In contrast, the T-complex protein 1 subunits (CCT5 and CCT8), epididymal sperm-binding protein E12 (ELSPBP1), proteasome subunit α type-6, and binder of sperm 1 (BSP1) were predominately found in bull spermatozoa with lower fertility and freeze-thawing rates (72). In cattle bull, many studies have attempted to identify protein markers of sperm cryo-tolerance or freeze-thawed semen fertility by the quantifying seminal plasma proteins (73–75). These studies identified BSPs as negatively related to the freezing ability or fertility in sperm cell either in seminal plasma (76, 77). The sperm-enriched proteomes identified based on access code, regulation, location, and function in different mammals are shown in Table 1.

**Table 1:** The freeze-thawed sperm enriched proteomes identified based on access code, regulation, location, and function in different farm animals and could be evaluated as a cryo-tolerance biomarkers.

**Protein name**	**Protein symbol**	**Organism**	**Access code**	**Protein regulation**	**Location**	**Function during cryopreservation**	**References**
Dihydrolipo amidedehydrogenase precursor	DLD	*Sus scrofa*	P09622	Up	Mitochondria	Hyperactivation of spermatozoa during capacitation and acrosome reaction	(78)
Inositol-1(or 4)- Monophosphatase	IMPA1	*Bos Taurus/Sus scrofa*	P29218	Up	Cytosol	Key enzyme of the phosphatidylinositol signaling pathway	(78)
S100 calcium binding protein A9	S100A9	*Bos taurus*/*Sus scrofa*	P06702	Down	Cytosol	Ca2+ binding protein	(78)
Soluble adenylyl cyclase (sAC)	ADCY10	*Oryctolagus cuniculus*	Q8C0T9		Fibrous sheet	cAMP production	(79)
β1,4galactosyltransferase 1 (GalT)	B4GALT1	*Bos taurus*	P15535		Apical Region	ZP3 (N-acetyl glucosamine)	(80)
Cysteine rich secretory protein 1	CRISP1	*Bos taurus/Equus caballus/Sus scrofa*	Q03401		Equatorial segment in capacitated sperm	Sperm-Oolemma Penetration	(81)
Cysteine rich secretory protein 1	CRISP2	*Capra hircus/Bos taurus/Sus scrofa*	P16563		Inner acrosome membrane	Sperm-Oolemma Penetration	(81)
ADAM metallopeptidase domain 2	ADAM2	*Bos taurus*/*Oryctolagus cuniculus*	Q99965		Integral membrane protein	Sperm-Oolemma Penetration	(82)
ADAM metallopeptidase domain 3A	ADAM3	*Bos taurus/Sus scrofa*	Q62287		Integral membrane protein	Sperm-Oolemma Penetration	(83)
Tektin 1	TEKT1	*Bos taurus/Sus scrofa*	Q969V4	Down	Flagella	Flagella- related	(84)
Septin 4	SEPT4	*Bos taurus*	O43236	Down	Annulus	Flagella- related	(85)
Isocitrate dehydrogenase subunit α	IDH3A	*Bos taurus*	P50213	Down	Mitochondria	Energy- Related	(139)
Izumo sperm-egg fusion 1	IZUMO1	*Bos taurus/Capra hircus/Sus scrofa*	Q9D9J7		Sperm cell-surface protein	Fertilization	(64)
Prostaglandin D2 synthase	PTGDS	*Ovis aries*/*Sus scrofa/Bos Taurus*	O02853		Testis, epididymis and prostate	Male reproductive system	(71)
Outer dense fiber protein 2	ODF2	*Sus scrofa/Bos taurus*	Q6AYX5		Sperm tail outer dense fibers	Association- with semen freezability	(64)
Voltage-dependent anion channel 2	VDAC2	*Sus scrofa Bos taurus*	CAB94711		Testis	Semen freezability	(63)
Phosphatidylethanolamine-binding protein 1	PEBP1	*Bos taurus*	NP001028795		Spermatozoa	Related to conception	(86)
Seminal plasma protein PDC-109 precursor	BSP1	*Bos taurus*	NP001001145		Plasma membrane	Sperm capacitation	(86)
Sperm acrosome associated 1	SPACA1	*Sus scrofa/Bos taurus*	Q9HBV2		Sperm acrosomal membrane-associated protein	Association with sperm freezability	(87)
Epididymal sperm-binding protein 1	ELSPBP1	*Bubalus bubali*s/*Sus scrofa*	Q96BH3		Epididymal origin	Sperm fertility	(88)

### Could the Spermatozoa Transcriptomics Profiling Provide Some Inspirations?

The underline mechanisms behind the effect of cryopreservation on sperm characteristics are not entirely understood. Genes and mRNA stability, protein expression, and epigenetic content of spermatozoa are thought to be modulated during the freezing-thawing process. Though, Ostermeier et al. (89), trusted that transcripts were expressed during spermatogenesis and that resistant transcript are assisted the sperm in struggling against the injury persuaded by the freezing-thawing cycle, the other residents of sperm transcripts were promptly degraded in response to cryo-stimulus. Some constraints of their study were that the authors could not elucidate why some novel transcripts were present. Some transcripts were upregulated after the freezing and thawing cycle (89).

Cryopreservation can affect the expression of critical genes such as genes encoding α, and β inhibin are potential candidates as fertility markers because both are significantly associated with sperm acrosomal integrity and motility (90). The embryogenesis-linked BCL2 like 11 (*BCL2L11*), BRCA1, and DNA that repair linked full-length transcripts in fresh bull semen were found abundantly in spermatozoa and are associated with structural components of ribosomes, while the transcripts detected in the lowest amounts are connected with ion transporter activity (91). Xue-Bing (92) described the ribosomal protein L31 (*RPL31*), which belongs to the ribosome multipart and is situated in the 60S subunit of the ribosome, as being differentially expressed between fresh and frozen-thawed sperm. The authors concluded that the RPL31 gene could be among other growth regulation genes in early embryonic growth. Nonetheless, the high expression of *RPL31* in cryopreserved sperm may be a result of cold stress and demands further exploration. Sperms are susceptible to oxidative damage due to their high polyunsaturated fatty acid content. Hence, glutamate-cysteine ligase catalytic subunit (*GCLC*) gene regulation in freeze-thawed sperm could be a protective comeback of the sperm to cold shock and oxidation stress. Besides, we found in a preceding work that the protein glutathione transferase mu5 (*GSTM5*), a fellow of the glutathione metabolic pathways, was upregulated in freeze–thawed sperm (93).

The role of transcriptomes such as sperm motility cation channel sperm associated 1 (CATSPER1) and sperm associated antigen 1 (SPAG1) in fertility and development of sperm hyperactivated motility has been clearly demonstrated in infertile male candidates; the knockout studies indicated that these transcripts are indispensable for the structural integrity of sperm (94, 95). Chen et al. (96) discovered four novel genes (e.g., *R1A10, R1C4, R4A1*, and *R4D2*), in fresh and cryo-preserved bull spermatozoa, were differentially expressed, and sequence results declared all four genes are regulated by ncRNAs transcripts, which may play a significant role during the freezing-thawing cycle and require further study (96). Cytochrome c oxidase polypeptide 5 (*COX5 A*) and (*COXI1*) are essential for mitochondrial function (24, 97). During mammalian sperm and oocyte fusion, phospholipase C zeta1 (*PLCZ1*) and phospholipase C beta1 (*PLCB1*) monitor the calcium signaling and aid sperm activation. High levels of PLCZ1 were found in spermatozoa, which are associated with phosphatidylinositol-linked enhancement of oocyte maturation *via* Ca^2+^ oscillations (98). The freeze-thawed sperm enriched transcripts related to fertility and cryo-sensitivity identified with a gene symbol, gene name, and functions are shown in Table 2.

**Table 2:** The freeze-thawed spermatozoa enriched transcripts identified based on their functions, location, transcripts per million (TPM), and unique gene reads (UGR) and can be evaluated as freezability biomarkers in farm animals.

**Gene Symbol**	**Gene Name**	**Access Code**	**Function during cro-preservation**	**Organism**	**Location**	**TPM**	**UGR**
*PRM1*	Protamine 1	NM_174156	Sperm progressive motility	*Bos taurus/Sus scrofa*	Chromosome25/Chromosome 03/	8,659	120
*YWHAZ*	Tyrosine 3-monooxygenase/tryptophan 5-monooxygenase activation protein, zeta	NM_174814	Association with Y chromosome	*Bos taurus/Sus scrofa/Equus caballus*	Chromosome 14/Chromosome 04/Chromosome 09	3,050	84
*FABP1*	Fatty acid binding protein 1	NM_001443	Sperm metabolism	*Bos taurus/Sus scrofa*	Chromosome 11/Chromosome 03/	2,923	1,074
*SCP2D1*	Sterol-binding domain containing 1	NM_001040507		*Bos taurus/Equus caballus*	Chromosome 13/Chromosome 22	2,726	182
*THSD4*	Thrombo spondin type 1 domain containing 4	NM_001078030	Hydrolase, peptidase activity	*Bos taurus/Equus caballus*	Chromosome 10/Chromosome 01	1,961	2,506
*CHMP5*	Charged multi vesicular body protein 5	NM_001034682	Inhibit apoptosis	*Bos taurus/Sus scrofa/Equus caballus*	Chromosome 08/Chromosome 10/Chromosome 23	1,693	260
*NR2E3*	Nuclear receptor subfamily2 group E member 3	NM_001167900	Maintenance of proper cell function	*Bos taurus*	Chromosome 10/	1,610	1,241
*SV2C*	Synaptic vesicle glycoprotein 2C	NM_001192019	Positively regulates the releasable pool of secretory vesicles	*Bos taurus/Equus caballus*	Chromosome 10/Chromosome 14	1,518	2,592
*MGC137055*	Det1and ddb1 associated	NM_001077080	Oxygen binding and carrier activity	*Bos taurus*	Chromosome 19	1,434	74
*GTSF1L*	Gametocyte specific factor 1-like	NM_001079601	Spermatogenesis	*Bos taurus/Equus caballus*	Chromosome 13/Chromosome 22	1,416	155
*TOE1*	Target of EGR1, member1 (nuclear)	NM_001075594	Cellular signaling, growth and proliferation	*Bos taurus/Gallus gallus*/*Equus caballus*	Chromosome 03/Chromosome 08/Chromosome 02	1,359	1,743
*SLC16A7*	Solute carrier family 16 member 7	NM_001076336	Monocarbooxylic acid trans-membrane transporter activity	*Bos taurus*	Chromosome 05/	1,284	2,831
*MCOLN2*	Mucolipin 2	NM_001192734	Carbonate dehydratase activity and zinc ion binding	*Bos taurus/Equus caballus*	Chromosome 03/Chromosome 05	1,231	1,756
*UNC119*	Unc-119 lipid binding chaperone	NM_001034645	Role in the mechanism of photoreceptor neurotransmitter release through the synaptic vesicle cycle	*Bos taurus/Equus caballus*	Chromosome 19/Chromosome 11	1,136	790
*CXCR4*	C-X-C motif chemokine receptor 4	NM_174301	Chemokine activity and heparin binding	*Bos taurus*	Chromosome 2/	1,095	975
*PAG5*	Pregnancy-associated glycoprotein 5	NM_176616	Aspartic-type endopeptidas activity	*Bos taurus/Ovis aries/Capra hircus*	Chromosome 29/Chromosome 21/Chromosome 13/	971	962
*MMP2*	Matrix metallopeptidase 2 (gelatinase A, 72-kDa gelatinase, 72-kDa type IV collagenase)	NM_174745	Stimulating Ca_2_+ ATPase activity	*Bos taurus/Sus scrofa*	Chromosome 18/Chromosome 06	933	1,417
*ITPA*	Inosine triphosphatase (nucleoside triphosphate pyrophosphatas)	NM_001076282	Chromosome organization	*Bos taurus/Equus caballus*	Chromosome 13/Chromosome 22/	919	458
*CCDC181*	Coiled-coil domain containing 181	NM_001205801	Coiled-coil proteins are important for the function of the centrosome, and help cell division	*Bos taurus/Capra hircus/Sus scrofa*	Chromosome 16/Chromosome 16/Chromosome 04	919	144
*DNAJB12*	DNAJ heat shock protein family (Hsp40) member B12	NM_001017946	Regulate molecular chaperone activity by stimulating ATPase activity	*Bos taurus/Bos indicus*	Chromosome 28/Chromosome 28/	914	2315

### The Potential Metabolomics Profiling and Sperm Cryo-Tolerance

A wide range of metabolomics biomarkers have been identified in sperm cells from boars (99), bulls (100), and goats (101), and these studies indicated that seminal fluid and spermatozoa metabolites might suggestively be connected to male breeding capability. The metabolites are assessed through developmental biological studies and thereby serve as metabolomics markers. In mouse sperm, the role and interaction of glycolytic metabolites with tyrosine phosphorylation were analyzed, whereas the outcome of this interaction is energy production which is vital for sperm freeze-thawed viability and motility (102). Amino acids play important roles in cellular physiology while also participating in the crucial phase of sperm cryobiology (103). In ram sperm, amino acid provides protection and regulation of metabolic activity and protects spermatozoa during cryopreservation, thereby decreasing lipid peroxidation and injury caused by free radicals (104).

In the meanwhile, carbohydrates are also present in the seminal plasma of animals and solely utilized in spermatozoa energy metabolism pathway (105). Spermatozoa consumed the surrounding seminal nutrients available in semen plasma and that nutrient metabolites, one way or another, control the signaling pathways and elaborate in spermatozoa hyperactivation, motility, capacitation, acrosomal reaction, freeze-thawing cycle, and spermatozoa–oocyte combination (106). Spermatozoa can be genetically (e.g., transcription and translation events) switched off, but metabolically is always switched on (107). The metabolic biomarker like “2-oxoglutarate aminotransferase” was mainly spotted in the boar spermatozoa (108), and was significantly found in low viable freeze-thawed sperm (109). The bioinformatics tools showed that metabolic pathways are playing an essential role in sperm cryopreservation, and hereby include the following pathways–citrate cycle “TCA cycle,” gluconeogenesis, dicarboxylate metabolism, glyoxylate, pyruvate metabolism, and galactose metabolism (110).

### Single Nucleotide Polymorphisms Markers Can Be Used for the Study of Sperm Cryo-Tolerance

The genome-wide association studies (GWAS) observed a sequence variation in the genome so-called SNPs, together with the pedigree and phenotypic evidence, thereby performing an association analysis and identifying genes or regulatory omics element that are significant for the trait of interest. GWAS approaches are much needed and practical in humans while also required in farm animals to develop SNPs biomarkers for phenotypic traits (111). Hering et al. (112) conducted the GWAS study upon high and low semen motility of Holstein bulls groups and identified the candidate gene *INCENP*, which is closely located to SNPs markers (rs109416157), associated with semen freezing quality. The progressive advancement in OMICS techniques made possible to measure the link between gene polymorphism and sperm freeze-thawed activity. RNA-Seq datasets were used to identify SNPs, and a total of 40 SNPs were genotyped, while several polymorphisms in *MS4A2, MAP3K20*, and *ROBO1* genes were significantly associated with sperm motility, membrane integrity, reduced cryo-induced lipid per-oxidase, and DNA damage in the boar spermatozoa (17). The genotyping frequencies are different among the genotype groups, while the Gene Ontology terminology (e.g., stress response) is relevant to polymorphisms, such as *MAP3K20* (rs340643892), *APPL1* (rs339379734), and *MS4A2* (rs339836492) and play an important role in the cryopreservation stresses (113). Different reports and evidence highlighted that polymorphisms in boar spermatozoa could be used as SNPs markers for semen quality (114). Nikbina et al. (137) performed a molecular experiment and analyzed the four SNPs related to caprine LHβ genes in exon3; these markers regulate the fresh and freeze-thawed semen quality characteristics of the boar. The most powerful SNPs such as FSHβ3 SNPs, FSHβ3-c, and FSHβ loci polymorphisms have been tested and investigated by Dai et al. (56) in semen freeze-thawed consistency characteristics and libido in goats. The results were consistent with previously available reports showing the impact of (FSHβ3) SNPs on semen quality in cattle bulls (56). Five SNP markers have been identified and are closely correlated with sperm freeze-thawed consistency and possible GnRH gene polymorphism in Chinese water buffalo. An association study found that g.3424T > C and g.3462C > A were used as high ejaculate volume markers, while g.991T > C, g.1041T > C, g.3424T > C, and g.3462C > A were used for decreasing sperm abnormality markers (115). Although evidence is present among the 3-UTR variants of the targeted mRNA, an association with semen quality has been shown (116).

## Bioinformatics Tools for Cryo-Markers Discovery

### Transcriptomic Tools

Advances in bioinformatics techniques have made it possible to isolate high-quality RNA from sperm and to develop novel non-invasive approaches to evaluate cryo-tolerance and post-thawed quality biomarkers in animals (86, 117).

Spermatozoa contain a subset of RNAs, including mRNAs, non-coding RNAs [ncRNA including microRNAs (miRNAs)], mitochondrial (mtRNA), and ribosomal RNAs (rRNAs) that can be routinely isolated from the sperm of several species including bulls, horses, and pigs (16, 72, 118). This novel approach is based on sperm RNA-sequencing (RNAseq) data analysis, by comparing the mRNA profile between higher and lower post-thawing semen to identify marker genes for mammalian semen post-thawing (16, 17).

The bovine spermatozoa transcript profile remains incomplete because previous studies have relied on hybridization-based techniques and did not provide information about full-length transcripts. In contrast, RNA-Seq studies based on high-throughput sequencing technology can assemble complete transcript sequences, including full-length mRNAs, and identify novel splicing junctions (119–121). RNA-seq (e.g., Illumina RNA-seq), using high-throughput next-generation sequencing (HT-NGS) technology that provides more excellent resolution for transcriptome profiling compared with other microarray technologies (122) and can identify candidate genes associated with more or less cryo-tolerant sperm. Gene annotation and gene analysis enable the researchers to investigate the genes relevant to multiple spermatozoa functions. Furthermore, the multiple candidate genes need to be validated for their link with high semen cryopreservation potential (61).

Differentially expressed genes are validated by quantitative real-time PCR (qRT-PCR), whereas the KASP™ assay analyzes SNP biomarkers. Combined studies of the transcriptome and proteome provide a clear picture of the genome, which could differentiate individuals likely to have high and low sperm cryo-tolerance (64). Microarray technology has been used to study the molecular mechanisms of spermatogenesis and the genomic etiology of male infertility. High-throughput technology has been effectively used for global gene profiling for mouse and bovine spermatozoa. A bovine oligonucleotide microarray (Affymetrix Bovine Gene-Chip) has been used to profile the transcript “fingerprints” of spermatozoa collected from high low-fertility bulls (117). Bioinformatics tools were used to select the differentially express genes and putative SNP markers potentially associated with good post-thawing and low post-thawing spermatozoa quality (113).

Next-generation sequencing (NGS) is the most reliable method to determine the small RNA profile in bull and pig spermatozoa. The sequencing of miRNAs and piRNAs in the semen of the bull was performed concerning different traits such as fertility, cryo-tolerance, and normal embryonic development (123).

### Proteomics Tools

High-throughput proteomic technology is especially useful to discover the biomarkers. Once the clinical value of proteomics markers are confirmed that it should be possible to develop the other cheaper tools, such as protein microarrays, mass spectrometry selective reaction monitoring (SRM), or multiplexed ELISA for routine biomarkers testing in the reproductive clinics (124). Proteins adenylate kinase isoenzyme1 (*AK1*), phosphatidyl ethanolamine-binding protein 1 (*PEBP1*), epididymal sperm-binding protein E12 (*ELSPBP1*), and binder sperm1 (*BSP1*) were noticed abundantly in the spermatozoa from the bulls with higher artificial insemination (AI) fertility rates and confirmed their differential expression by Western blotting analyses. Moreover, a linear regression model was also used to determine the link between the fertility rate and protein abundance. This model investigated proteins like such as CCT5 and AK1, both of which influence spermatozoa cryo-tolerance and fertility rates in higher AI rates (88). Mass spectrometry-based targeted proteomics approaches such as selected reaction monitoring are developed as a gifted tool for the verification of candidate proteins in biological and biomedical submissions. The unbiased “discovery” proteomics examination, e.g., “shotgun' proteomics,” can now deliver genome-scale coverage and quantification of both proteomics and post-translational modifications using extensive fractionation and stable isotope labeling (125).

Differential labeling followed by the LC-MS/MS technique was used to carry out proteomic analysis, and high numbers of differentially expressed proteins were identified in asthenozoospermia patients. The other non-proteomic techniques such as *ELISA*, immunofluorescence, enzymatic activity, flow cytometry, immunochemistry, and Western blotting were also used to detect differentially expressed sperm protein (126–128). More significantly, proteins are the primary driving force in almost all cellular developments. Hence, protein microarrays were established as a high-throughput apparatus to overcome the constraint of DNA microarrays and provide a direct platform for protein function analyses. At about the same time, an additional protein microarray was settled through the immobilization of purified proteins on glass slides. To discriminate this type of array from the antibody arrays, they are separated into analytical and functional (138).

### Lipidomics and Metabolomics Tools

Like all tiny molecules, lipids are produced and metabolized by enzymes that are influenced by the environmental factors of a given biological system, for instance, by the nutrition and temperature. Initial reports of mass spectrometric analysis using soft ionization techniques such as matrix-assisted laser desorption ionization (MALDI) and electrospray ionization of multifaceted lipid mixtures were published by Wenk (129). The foremost objective of lipidomics is the complete classification of different lipid species and their natural roles concerning the expression of proteins involved in lipid metabolism and function, including gene regulation (130). Lipidomics is relatively a new area of research that has seen rapid progress in analytical technologies such as mass spectrometry (MS), fluorescence spectroscopy, dual-polarization interferometry, spectroscopy of NMR, and computational methods that help the identification of the position of molecular species of lipids (131). The phospholipids and fatty acid configurations of boar spermatozoa for cryo-resistance are compared by using matrix-assisted laser desorption and ionization time-of-flight mass spectrometry (MALDI-TOF MS) in combination with thin-layer chromatography and 31P NMR spectroscopy. Metabolomics techniques like NMR and GC-MS have been widely used to identify possible biomarkers for freeze-thawed sperm fertility in cattle bulls (132) and men (133–135). Two well-known techniques are used to study metabolomics biomarkers on a wide range, MS-based methods and NMR spectroscopy-based methods. Organic acids, carbohydrates, amino acids, and lipid anti-oxidants are the major metabolites in seminal fluids, and these classes were measured by spectrophotometric, colorimetric, and thin-layer chromatography methods such as “TLC” and NMR. High-resolution proton NMR spectroscopy has proved to be one of the most potent bio-fluid and intact tissue analysis technologies, providing a wide-ranging profile of metabolite signals without isolation, derivatization, and pre-selected parameters of measurement (136).

## Conclusions and Future Perspective

The OMICS profiling data from various spermatozoa freezability groups, in combination with advanced bioinformatics technology consisting of Illumina RNA-seq, high-throughput next-generation sequencing (HT-NGS) technology, multiplexed ELISA, should be used to identify the routine biomarkers for good and poor cryo-tolerance farm animals. Combining these powerful technologies would provide a deeper insight into the molecular and cellular changes induced by the freezing-thawing process, and would allow data analysis in different cryopreserved samples to determine the spermatozoa freezing capacity of farm animal species. Besides, a validation technique is required to approve whether candidate genes and putative SNP markers may contribute to high cryo-tolerance of sperm. This useful knowledge, which has been extensively presented in this report, is important for the identification of potential biomarkers to predict spermatozoa freezability more accurately and for the development of new policies to improve the results of cryo-preserved spermatozoa. Nevertheless, systematic analysis of the specific genetic markers that may facilitate the post-thawing cycle would be a feasible approach to distinguish a male breeding stock, which has the excellent genetic potential for cryopreservation. A long-lasting follow-up study on the subsequent offspring obtained from good cryo-resistance freeze-thawed spermatozoa is suggested for future works.

## Author Contributions

IK and YZ: conceptualization. SR: formal analysis and software. YZ, ZC, and HL: funding acquisition. IK, AK, and MK: investigation. IK: writing original draft. YZ: project administration. HL: resources. ZC: visualization. IK, YZ, and AS: writing review and editing. All authors contributed to the article and approved the submitted version.

## Conflict of Interest

The authors declare that the research was conducted in the absence of any commercial or financial relationships that could be construed as a potential conflict of interest.
